# Effect of scan strategy on the formation of a pure nickel single-crystal structure using a flat-top laser beam via laser powder bed fusion

**DOI:** 10.1080/14686996.2023.2201380

**Published:** 2023-04-25

**Authors:** Dennis Edgard Jodi, Tomonori Kitashima, Makoto Watanabe

**Affiliations:** a Research Center for Structural Materials, National Institute for Materials Science, Tsukuba, Japan; b Department of Materials Physics and Chemistry, Kyushu University, Fukuoka, Japan; c Department of Materials, Kyushu University, Fukuoka, Japan

**Keywords:** Metallic materials, crystal growth, single crystal, additive manufacturing

## Abstract

In this study, the role of a scan strategy in the fabrication of a single crystal (SX) structure of face-centered cubic (fcc) pure Ni using a flat-top laser profile by a laser powder bed fusion (LPBF) process was investigated. A flat-top laser with a uniform heat intensity across the beam profile was employed to fabricate the SX structure with a <001>  texture parallel to the build direction (║BD). A meander scan strategy with an XY–90° scan rotation was required to achieve the flat-top-derived SX structure. The meander scanning strategy promoted the epitaxial <001> fcc growth along the direction ║BD, leading to the <001>║BD texture formation. Meanwhile, the XY–90° scan rotation induced the epitaxial growth of <001> fcc phase on the hatch direction (HD)–scan direction (SD) plane in the direction deviated by approximately 45° relative to the SD. This 45° deviation relative to the SD occurred to accommodate the beam movement and the circular beam geometry on the HD – SD plane, resulting in the preferred configurations of <011>║SD and HD. Thus, this paper reports the importance of meander scanning with a 90° scan-rotation strategy to yield a flat-top-LPBF-derived SX structure with <001>║BD and <011>║SD and HD textures.

## GRAPHICAL ABSTRACT

**Figure:**
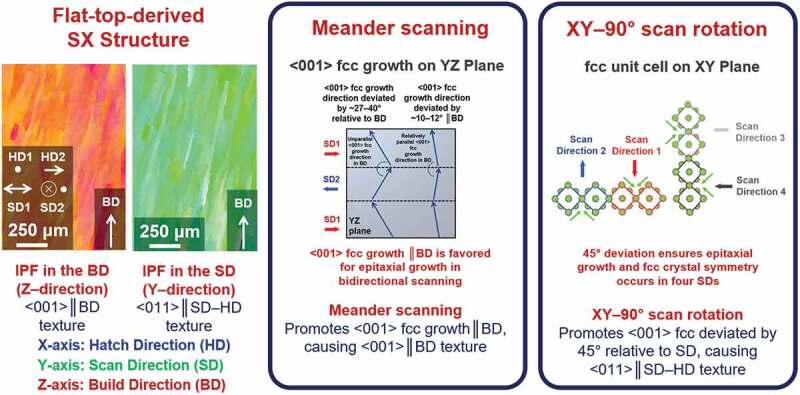

## 1. Introduction

A recent trend in laser powder bed fusion (LPBF) research has confirmed the feasibility of fabricating net-shaped products in a time- and cost-effective manner. Various techniques have been employed to control the microstructure of LPBF products. These techniques include adjusting the laser power, scan speed, hatch space, or scan rotation among other scanning parameters [1,2] or utilizing various laser beam profiles to melt the powder bed [3]. The implementation of different laser beam profiles, particularly by adopting a flat-top profile [4], prevented the formation of defects, such as keyhole pores, due to the weak melt flow and reduced vortex flow velocity in the molten melt pool [5]. The flat-top laser beam exhibits a flat intensity profile over the beam spot area, generating the uniform energy distribution [3,6–8], whereas the Gaussian laser beam has an intensity profile decayed from its maximum at the spot center to zero along the beam radius [6]. Furthermore, the uniform heat-intensity in the flat-top profile has yielded promising results in achieving homogeneous textures [3,9,10] and single crystal (SX) structures [11]. The uniform heat intensity distribution in the flat-top laser resembles the heat distribution of an electron beam in an electron beam powder bed fusion (EPBF) process, which has been studied to cause a <001> ║BD texture and an SX structure [12–15]. However, the fabrication of an SX structure by LPBF using the typical Gaussian profile is challenging because of the focused heat intensity at the center of the beam area [16]. The concentrated heat intensity in the Gaussian beam caused the maximum thermal gradient in the lateral direction at the melt pool edge, resulting in the deviated <001> fcc growth by 45° relative to BD [17]. The 45° deviation of <001> fcc growth relative to BD then generated typical <011>║BD texture in the melt pool edge of Gaussian-derived specimens [17]. At the melt pool center, however, a vertical thermal gradient direction was parallel to BD and it caused the formation of <001> fcc growth in the direction ║BD and <001>║BD texture in the Gaussian-derived melt pool center. Therefore, the heterogeneous texture formation in the Gaussian-derived melt pool has been challenging to yield SX structure formation [16].

An SX structure is typically obtained by directional solidification (DS) [18,19]. In the DS process, a low cooling rate at a few inches per hour is employed, which induces a high thermal gradient-to-cooling rate (*G*/*R*) ratio and promotes epitaxial planar grain growth, affording the SX structure [20]. Consequently, obtaining an SX structure via the DS process is time-inefficient because of the low cooling rate. Additionally, primary and secondary grain selections in the DS process are necessary to promote the uniform growth of the face-centered cubic (fcc) lattice in the planes parallel and perpendicular to the cooling direction [18,19]. For fcc-based metals and alloys, such as pure Ni, the grains are solidified favorably in the <001> direction parallel to the thermal-gradient direction [20]. The unidirectional cooling in the DS process causes the <001> fcc growth to occur parallel to the cooling direction during solidification, i.e. primary grain selection [20]. In contrast, the secondary grain selection controls the <001> fcc growth direction on the plane perpendicular to the cooling direction, as contributed by the geometrical blocking mechanism in the grain selector [21]. The geometrical blocking mechanism is influenced by the thermal-gradient direction in the grain selector. Wang et al. [18] reported that controlling the thermal-gradient direction in the grain selector is challenging. This control issue causes the random selection of the <001> fcc growth direction on the plane perpendicular to the cooling direction. In other words, the DS process can yield products with different textures even from the same mold design [18].

In the LPBF process, a high *G*/*R* ratio favors epitaxial solidification in the melt pool [22]. The high *G*/*R* ratio is induced by the high thermal gradient from the small melt pool relative to the surrounding powder bed [23]. The thermal gradient in the LPBF process can reach>10^7^ K/m, which is significantly beyond the range of 10^4^–10^5^ K/m in a typical DS process [16,24,25]. Other nucleation types, e.g. bulk or fusion boundary nucleation, require a low *G/R* ratio, significant undercooling, or a high density of precipitate or unmelted particles [22]. Thus, other nucleation types are not preferred unless high-temperature stage heating or precipitate-containing alloy systems are utilized [22,26].

However, a different grain-selection process was suggested in the LPBF and EPBF processes because of the lack of grain selectors for the SX structure formation [11–15,27]. Fernandez-Zelaia et al. [13] reported that adjusting the local heat transfer and solidification dynamics, which are controlled by the following scanning parameters: the line offset, scan speed, and scan current, promoted epitaxial growth during the EPBF-based SX structure with misorientation tolerance of 7.5°. Further, the importance of controlling the scanning parameters has been reported by Chauvet et al. [14], i.e. tuning the melting parameters is necessary to control the thermal gradient and growth rate to obtain an EPBF-derived SX with an epitaxial columnar grain structure with misorientation tolerance of 15°. However, the above studies [13,14] do not disclose how adjusting the scanning parameters can lead to grain selection for the SX structure formation. Meanwhile, Gotterbarm et al. [15] highlighted that the scan strategy affects the SX structure formation. Implementing a μ-helix scanning strategy, which mimics the spiral grain selector in the DS process, causes the mean thermal-gradient direction to be parallel to the build direction (║BD). This favors the growth of <001> fcc ║BD and leads to the <001>║BD SX structure formation [15]. However, their assumption does not apply to the SX structure in [11], which was fabricated by a conventional XY–90° rotation. Additionally, the <001> fcc growth in the scan direction (SD) and hatch direction (HD) axes is reported to be deviated by 45° relative to the SD and HD axes, forming <011>║SD and HD textures in the EPBF-derived SX structures [13–15]. To date, the grain-selection process for the <001> fcc growth in the SD and HD axes, as well as the formation of <011>║SD and HD textures in the EPBF-derived SX structures, has not been clearly understood, as plainly stated in [13–15]. Pistor et al. [12] proposed that the <011>║SD and HD textures in the EPBF-derived SX structure were induced by the {111} <110> fcc slips during the plastic tension – compression deformation. However, the proposed theory in [12] contradicts the result in [11], as the LPBF-derived SX structure does not display significant plastic deformation, which is indicated by the suppressed strain accumulation and low dislocation density. Thus far, the comprehension of the flat-top-LPBF-derived SX structure formation is limited to controlling the melt-pool geometry to realize the <001> fcc growth in the direction ║BD [11]. Thus, another mechanism might be responsible for the flat-top-LPBF-derived SX structure formation.

This study investigates the contribution of a scan strategy to the formation of a pure Ni flat-top-LPBF-derived SX structure. Furthermore, although the SX structure has been successfully fabricated using EBPF, only conductive metal or alloy powders can be melted in EPBF whereas the absorption efficiency of the electron beam in the powder bed is independent on the type of metals in EBPF [28]. Thus, achieving an SX structure via the flat-top-derived LPBF process affords increased flexibility in alloy types or design geometries for future mass production. Expectedly, this study will improve the understanding of the influence of scan strategies on the SX formation mechanism in a flat-top-derived LPBF process.

## 2. Experimental procedures

**Figure 1.**
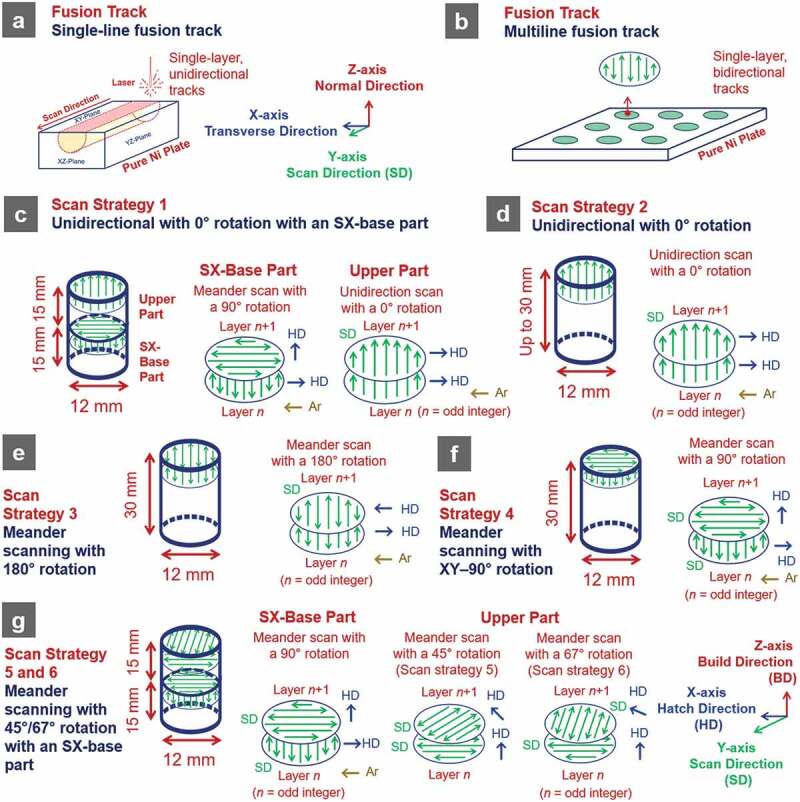
Schematic of the scan strategy in this study; (a) single-line and (b) multiline fusion track; (c) scan strategy 1, (d) 2, (e) 3, (f) and 4 and (g) 5 and 6 for the multilayer specimens.

The fabrication was performed using an SLM Solutions SLM280HL system (Germany) in an inert Ar atmosphere. The LPBF apparatus was equipped with a flat-top beam profile with a beam diameter of 700 μm. The beam profile of a flat-top laser in the SLM280-type machine was represented elsewhere [7]. Before the fabrication, melt-pool analysis was conducted using fusion tracks on a polycrystalline pure Ni plate. The fusion track experiment was conducted at a laser power (*P*) and scan speed (*v*) of 500 W and 140 mm/s, respectively, and the schematic of the fusion track is displayed in Figures 1(a,b). The XZ and XY planes of the single-line fusion track were observed (Figure 1(a)), whereas multiline tracks were employed for the YZ-plane observation because the width of a single-line track was small (Figure 1(b)). Several additional fusion track experiments at *P* of 500 W and *v* between 25 and 220 mm/s were also carried out. Pure Ni powder was not coated for the fusion-track experiments. The melted zone was qualitatively determined by distinguishing the area with elongated grains or non-equiaxed grain structures, from the equiaxed-structure. This method is helpful in analyzing the melted zone of pure metals in LPBF studies [1,11].

**Figure 2.**
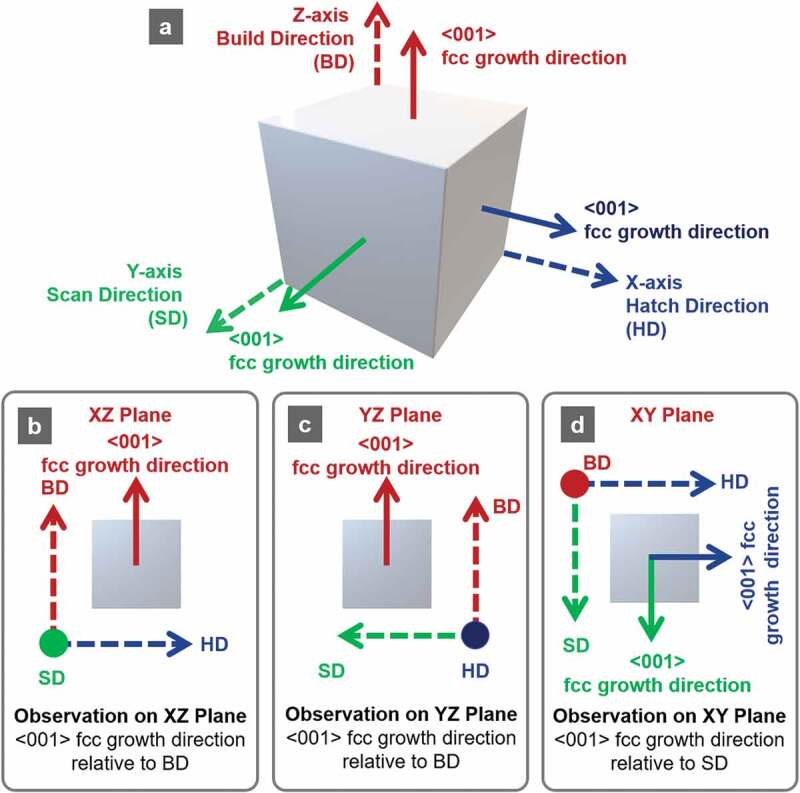
(a) Schematic of the X–, Y–, and Z–directions in this study; schematic of the <001> fcc direction.

Following the fusion-track observations, full-scale multilayer specimens were fabricated on polycrystalline stainless steel 304 substrates. The pure Ni powder exhibited *D*_10_, *D*_50_, and *D*_90_ sizes of approximately 24, 34, and 51 μm, respectively. The parameters for the multilayer specimens were *P*–*v* of 500 W–140 mm/s, with a hatch space (*h*) of 100 μm. The energy density (*E*_*d*_) of the multilayer parameter in this study was 43.07 J∙mm^−3^ following the postulation by Ferro *et al* [29]. as,

(1) $$E_{d}=\frac{\beta P}{h\sqrt{4\alpha d_{b}v}},$$

where β, and α are absorption factor (approximately 0.8 [30]) and thermal diffusivity (approximately 22 mm^2^/s for pure Ni [31]), respectively. The layer thickness was 30 μm, and the scan strategy design is illustrated in Figure 1(c–g). In scan strategy 1 (Figure 1(c)), the pure Ni powder was deposited until a 15-mm building height was realized using the SX – yielding scan strategy of specimen FT1 in [11], where FT1 was fabricated using a flat-top laser beam with *P-v-h* of 500 W–140 mm/s–100 μm scanned by meander scanning and XY–90° scanning rotation [11]. The next 15-mm building height was fabricated by unidirectional scanning (green arrows in Figure 1(c))) with a 0° rotation (blue arrows in Figure 1(c)). The lower part with an SX structure is called ‘SX-base part’ in this study as shown in Figure 1. In scan strategy 2 (Figure 1(d)), the pure Ni powder was deposited using unidirectional scanning (green arrows in Figure 1(d)) with a 0° rotation (blue arrows in Figure 1(d)) until a 30-mm building height was realized. In scan strategy 3 (Figure 1(e)), a bidirectional meander scanning strategy (green arrows in Figure 1(e)) was employed, followed by a 180° rotation (blue arrows in Figure 1(e)) to fabricate 30 mm-height specimens. In scan strategy 4 (Figure 1(f)), a meander scanning strategy (green arrows in Figure 1(f)) was employed, followed by an XY–90° rotation (blue arrows in Figure 1(f)) to fabricate 30 mm-height specimens. For scan strategies 5 and 6, the SX – yielding scan strategy (FT1 in [11]) was used until a 15-mm building height was achieved. Afterward, meander scanning (green arrows in Figure 1(g)) with 45° (scan strategy 5) or 67° (scan strategy 6) scan rotations was performed in each layer (blue arrows in Figure 1(g)) to build the remaining 15-mm height. The scan strategies in this study are summarized in Table 1. The X, Y, and Z axes represent HD, SD, and BD, respectively (Figure 2(a)). The Ar gas flow direction is indicated in the applicable figures (Figures 4–7) because it has been demonstrated that the Ar gas flow affected the crystal texture formation [32] although it is a future work to investigate the effect of the gas flow on the texture formation in this study. Stage heating was not adopted in this study.

**Table 1.** Scan strategies adopted in this study.

Scan strategy number	Height of fabricated part(s)	Scan direction	Scan rotation
1	Upper 15 mm (Upper part)	Unidirectional	0°
	Lower 15 mm (SX-base part)	Meander	XY-90°
2	30 mm	Unidirectional	0°
3	30 mm	Meander	180°
4	30 mm	Meander	XY-90°
5	Upper 15 mm (Upper part)	Meander	45°
	Lower 15 mm (SX-base part)	Meander	XY-90°
6	Upper 15 mm (Upper part)	Meander	67°
	Lower 15 mm (SX-base part)	Meander	XY-90°

After the fabrication, the multilayer specimens were removed from the stainless steel build substrate via a wire-cutting process. The fusion track and multilayer specimens for scan strategies 1–3 were cut parallel to the XZ (Figure 2(b)), YZ (Figure 2(c)), and XY (Figure 2(d)) planes to reveal the crystal structures in the SD, HD, and BD axes, respectively. The multilayer specimens for scan strategy 4 were cut parallel to the XZ/YZ planes and XY planes to reveal the crystal structures in the SD/HD and BD axes, respectively. Regarding the specimens for scan strategies 5 and 6, the cross-sections were cut parallel to the BD axes, considering that the SD and HD axes were not clearly defined because of the 45°/67° scan rotation performed. The <001> fcc growth direction was influenced by the thermal-gradient directions relative to the BD and SD [33]. Thus, in this study, the <001> fcc growth direction was observed relative to the BD and SD, as illustrated in Figure 2(b–d). The surface preparation was conducted using abrasive papers (grit #320 and #600), diamond suspensions (9, 3, and 1 µm), and a colloidal silica suspension (0.25 µm). The microstructure was analyzed using a scanning electron microscope with an attached electron backscattered diffraction detector (SEM – EBSD, JEOL JSM-7001F (Japan)). The EBSD observation in the XZ and YZ planes covered an area of 800 × 4000 µm with a step size of 5 μm. Further, the EBSD data in the XY plane were obtained in an area of 400 × 400 µm with a step size of 1.2 μm. A more detailed observation of the SX structure (as shown in Figures 9 and 10) covered an area of 1000 × 1500 µm with a step size of 4 μm. The EBSD data were analyzed using the TSL OIM 7 software. The texture of the multilayers specimens was analyzed three-dimensionally on the XZ, YZ, and XY planes. The inverse pole figure (IPF) maps for the texture orientation analysis were set in the HD (X – direction), SD (Y – direction), and BD (Z – direction).

## 3. Results

### 3.1. Three-dimensional characterizations of the melt-pool geometry

**Figure 3.**
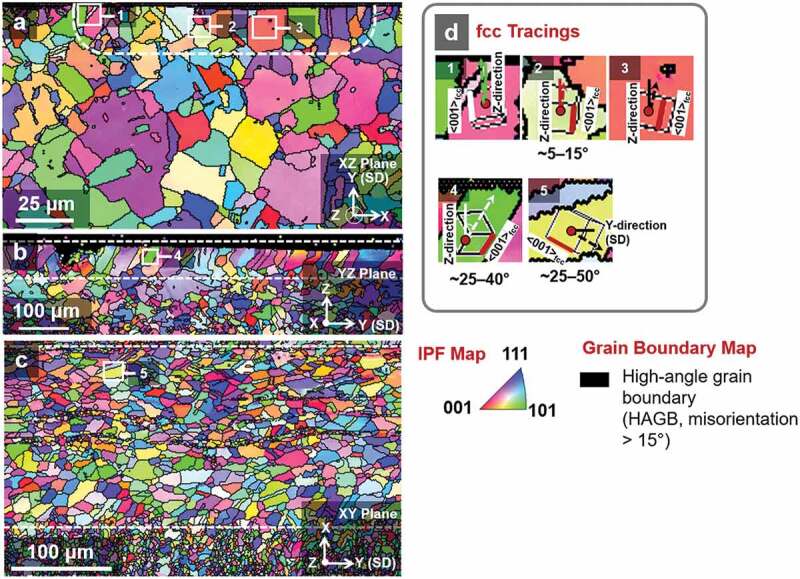
Inverse pole figure (IPF) maps in the Z–direction for the fusion track observations; (a) XZ, (b) YZ, and (c) XY planes; (d) fcc tracings from the respective numbered points; white dashed lines indicate the estimated melt-pool area.

The melt-pool geometry shown in Figure 3 was obtained from the fusion tracks with the *P*–*v* of 500 W–140 mm/s. The Y – direction in the fusion track in Figure 3 represented the SD, whereas X – and Z – directions in Figure 3 represented transverse and normal directions. The melt pool was analyzed three-dimensionally for comparison with the multilayer observation results.

#### 3.1.1. The <001> fcc growth direction relative to the normal direction (Z–direction) in the XZ plane

The <001> fcc growth direction relative to the BD in the XZ plane (Figure 2(b)) was influenced by the beam profile’s intensity distribution and the laser processing parameters, e.g. laser power or scan speed [34]. Controlling the <001> fcc growth direction relative to the BD in the XZ plane has been reported to be one of the beneficial aspects of achieving an LPBF-derived SX structure [11]. The <001> fcc growth direction on the XZ plane in the fusion track analysis (Figure 3(a)) was closely ║normal direction across the melt pool (red lines in tracings 1–3 in Figure 3(d)), showing an approximate deviation of 5°–15° relative to the normal direction. The <001> fcc growth in the direction ║normal direction in the XZ plane, which was achieved by optimizing the laser power and scan speed parameters, suggested a relatively uniform heat-intensity distribution across the melt-pool area.

#### 3.1.2. The <001> fcc growth direction relative to the normal direction (Z–direction) in the YZ plane

The characterization of the fusion-track specimens in the YZ plane revealed the <001> fcc growth direction relative to the BD in the YZ plane (Figure 2(c)). Figure 3(b) displays a solidification structure deviated by approximately 25°–40° relative to the normal direction near the top area on the YZ plane. Tracing 4 in. 3d suggests that the <001> fcc growth direction in the YZ plane deviated by approximately 34° relative to the normal direction. This indicates that although the <001> fcc growth direction was relatively ║normal direction in the XZ plane, the actual <001> fcc growth direction deviated relative to the normal direction on the YZ plane. The distribution of the <001> fcc growth direction in the XZ plane was dependent on the laser power – scan speed combinations or laser profile [11]. However, the deviation of the <001> fcc growth direction relative to the BD in the YZ plane was caused by the beam scanning movement, as demonstrated in previous studies [35–37].

#### 3.1.3. The <001> fcc growth direction relative to the SD (Y–direction) in the XY plane

The characterization in the XY plane revealed the <001> fcc growth direction relative to the SD in the XY plane (Figure 2(d)). Controlling the <001> fcc growth direction in the XY plane is necessary to achieve an additively manufactured SX structure, as previously reported [38]. The elongated and tilted grain structure shown in Figure 3(c) was not observed because of the single-line melting. However, the tracings, e.g. tracing 5 in Figure 3(d), suggested that the <001> fcc growth direction in the XY plane was deviated by approximately 25°–50° relative to the SD. The deviation was induced by the beam movement of the circular beam geometry, as will be further discussed in the Discussion section.

### 3.2. Three-dimensional characterizations of the multilayer specimens

The multilayer specimens were fabricated using the fusion track parameters with a hatching space of 100 μm. The overlap ratio between beads in this study was approximately 35%, which was calculated from the melt pool width of approximately 150 μm and the hatch space of 100 μm. The gap in the melt pool widths and the beam diameter is also caused by the scanning-parameter difference, e.g. laser power or scan speed, as has been observed in previous studies [8,39–41]. The flat-top-derived melt pool in the fusion track represented the <001> fcc growth in the direction ║BD in the XZ plane, as shown in Figure 3(a). The characterization of the multilayered specimens was conducted three-dimensionally to observe the effects of the scan strategy on the texture formation, <001> fcc growth direction on each plane, and SX structure formation.

#### 3.2.1. Texture configurations in the SD (Y–direction) and <001> fcc growth direction relative to the BD (Z–direction) in the XZ plane with variations in scan strategies

##### 3.2.1.1. Texture configuration in the SD (Y–direction) in the XZ plane

**Figure 4.**
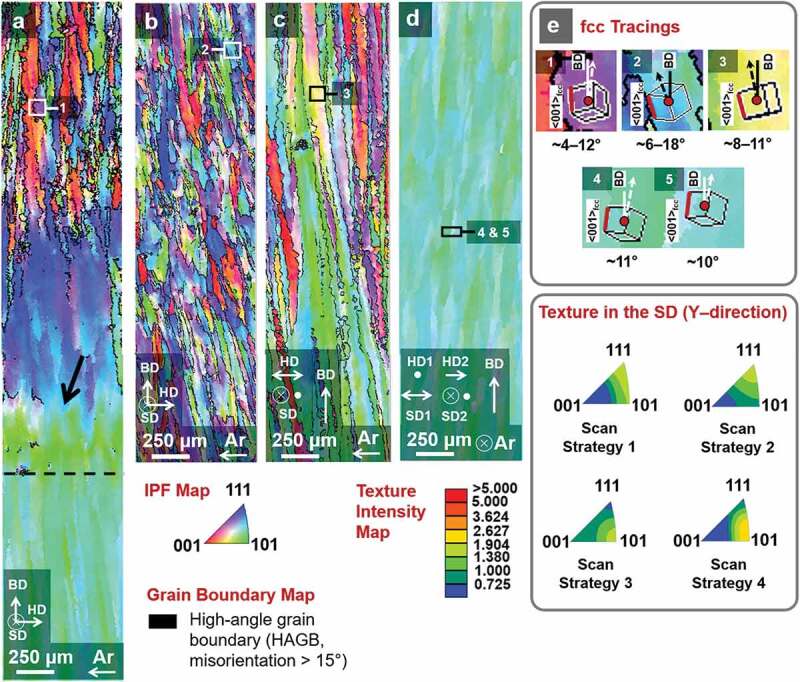
IPF maps in the SD (Y–direction) in the XZ plane for multilayers specimens with scan strategies (a) 1, (b) 2, (c) 3, and (d) 4; (e) fcc tracings from the respective numbered points; a black dashed line in (a) indicates the boundary between the upper and SX-base parts. For (d), the IPF maps were for the XZ/YZ plane attributed to the XY–90° scan rotation. The IPF maps were taken at (a) 14 mm and (b – d) 25 mm above the stainless steel build substrate.

Variations in scan strategies influenced the texture configuration in the XZ plane. Figure 4 shows the IPF maps of the upper part of the specimens with scan strategies 1 (Figure 4(a)), 2 (Figure 4(b)), 3 (Figure 4(c)), and 4 (Figure 4(d)). Although the upper and SX-base parts were fabricated using the same parameter in scan strategy 1, different scan strategies between the upper and SX-base parts caused different texture formations, as shown in Figure 4(a). The SX-base part displayed an SX structure with <011>║SD texture. In contrast, the upper part showed non-SX < 112>– <111>║SD texture by implementing unidirectional scanning with a 0° rotation. Scan strategy 2 (Figure 4(b)) revealed that the texture formed by the unidirectional scanning was maintained from the bottom until a relatively high building (e.g. 30 mm) was achieved. Implementing a meander scanning with a 180° rotation in scan strategy 3 (Figure 4(c)) partly changed the texture formation to <011>║SD. The texture change from <112>– <111>║SD to <011>║SD suggested that the <001> fcc growth direction relative to the BD was decreased by approximately 35°. Furthermore, the change in texture indicated that the meander scanning influenced the <001> fcc growth direction relative to the BD, as will be discussed in the following section. Further, the <011>║SD texture formation, along with an SX structure formation, (Figure 4(d)) was observed in the specimens scanned by meander scanning complemented by an XY–90° scan rotation (scan strategy 4).

##### 3.2.1.2. <001> fcc growth direction relative to the BD (Z-direction) in the XZ plane

The changes in the texture configuration and texture intensity suggested that scan strategies influenced the <001> fcc growth direction. The <001> fcc growth direction in the XZ plane (e.g. tracings 1–5 in Figure 4(e)) was relatively ║BD regardless of the scan strategies. In the unidirectionally scanned specimens with scan strategies 1 and 2, the <001> fcc growth direction was deviated by approximately 4°–18° relative to the BD (e.g. tracings 1 and 2 in Figure 4(e)). The deviation of the <001> fcc growth direction slightly decreased by implementing meander scanning at 8°–11° relative to the BD (e.g. tracing 3 in Figure 4(e)). Complementing the meander scanning with an XY–90° rotation in scan strategy 4 further decreased the deviation of the <001> fcc growth direction to 10°–11° relative to the BD (tracings 4 and 5 in Figure 4(e)).

#### 3.2.2. Texture configurations in the HD (X–direction) and <001> fcc growth direction relative to the BD (Z–direction) in the YZ plane with variations in scan strategies

##### 3.2.2.1. Texture configuration in the HD (X–direction) in the YZ plane

**Figure 5.**
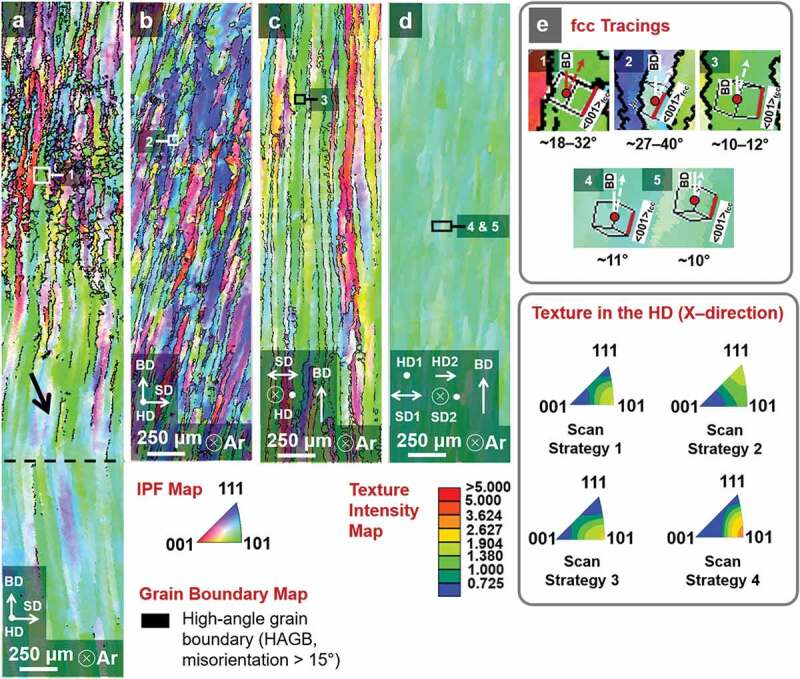
IPF maps in the HD (X–direction) in the YZ plane for multilayer specimens with scan strategies (a) 1, (b) 2, (c) 3, and (d) 4; (e) fcc tracings from the respective numbered points; a black dashed line in (a) indicates the boundary between the upper and SX-base parts. For (d), the IPF maps show the XZ/YZ plane attributed to the XY–90° scan rotation. The IPF maps were taken at (a) 14 mm and (b – d) 25 mm above the stainless steel build substrate.

Scan strategies influenced the texture formation in the YZ plane. The unidirectionally scanned upper part shown in Figure 5(a) displayed the formation of <011>– <112>║HD textures. In the unidirectionally scanned scan strategy 2, the texture changed to the <112>– <111>║HD in the high buildings (Figure 5(b)). Meander scanning in scan strategy 3 changed the texture to <011>║HD (Figure 5(c)), indicating the change in the <100> fcc cell growth direction relative to the BD, as discussed in the following section. Finally, the <011>║HD texture formed suppressing high-angle grain boundaries (HAGBs) with an SX structure (Figure 5(d)) by implementing meander scanning and XY–90° scan rotation in scan strategy 4.

##### 3.2.2.2. <001> fcc growth direction relative to the BD (Z–direction) in the YZ plane

The texture change from <112>– <111>║HD in the unidirectionally scanned scan strategy 2 to <011>║HD in the meander scanned scan strategies 3 and 4 occurred because of the change in the <001> fcc growth direction relative to the BD in the YZ plane (e.g. fcc tracings in Figure 5(e)). The <001> fcc growth direction in the unidirectionally scanned scan strategies 1 and 2 (e.g. tracings 1 and 2 in Figure 5(e)) deviated by 18°–32° and 27°–40° relative to the BD, respectively. Meanwhile, the meander scanning in scan strategy 3 reduced the deviation of the <001> fcc growth direction in the YZ plane toward 10°–12° relative to the BD (e.g. tracing 3 in Figure 5(e)). The deviation decrement by up to 30° was supported by the texture change from <011>– <112>║HD or <112>– <111>║HD in the specimens with scan strategies 1–2 to <011>║HD in the specimens with scan strategy 3. The <001> fcc growth direction in the YZ plane for scan strategy 4 was similar to that for scan strategy 3, displaying deviations of 10°–11° relative to the BD (tracings 4 and 5 in Figure 5(e)). The background of the <001>-fcc-growth direction change in the YZ plane with different scan strategies will be discussed in the Discussion section.

#### 3.2.3. Texture configurations in the BD (Z–direction) and <001> fcc growth direction relative to the SD (Y–direction) in the XY plane with variations in scan strategies

##### 3.2.3.1. Texture configuration in the BD (Z–direction) in the XY plane

**Figure 6.**
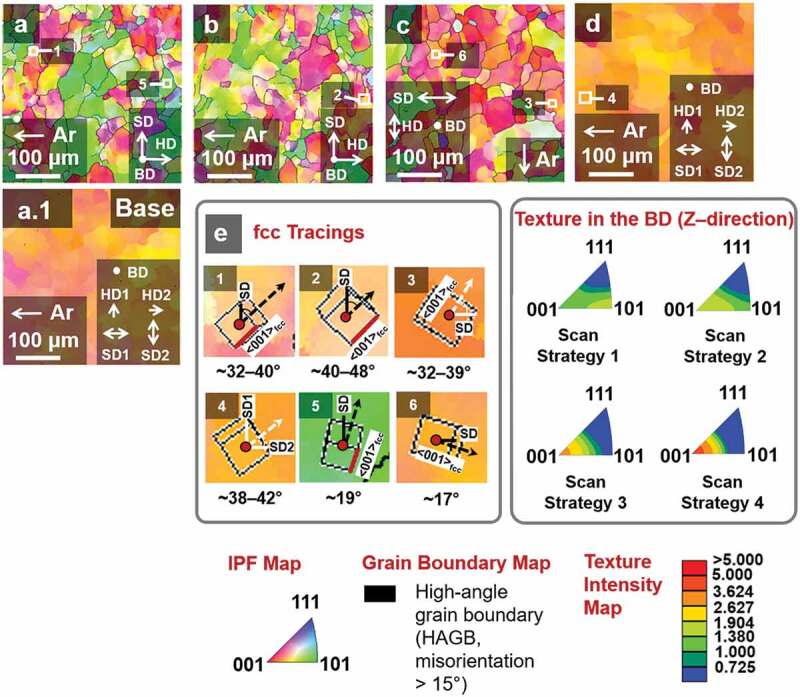
IPF maps in the BD (Z–direction) in the XY plane for multilayer specimens with scan strategies (a) 1, (b) 2, (c) 3, and (d) 4; (e) fcc tracings from the respective numbered points; Fig. a.1 displays the SX-base part for scan strategy 1 taken at 13 mm above the stainless steel build substrate. The IPF maps were taken at (a) 18 mm and (b – d) 30 mm above the stainless steel build substrate.

The influence of scan strategies on the texture formation in the XY plane is displayed in Figure 6. The textures in the unidirectionally scanned specimens, as shown in Figure 6(a,b), were dominantly configured as <001>– <011>║BD in the upper part of the specimen at 18 mm height with scan strategy 1 and for the top part with scan strategy 2. Implementing a meander scanning in scan strategy 3 (Figure 6(c)) induced a more prominent <001>║BD texture. The utilization of the meander scanning with an XY–90° rotation in scan strategy 4 (Figure 6(d)) induced a more enhanced <001>║BD, followed by the elimination of the HAGB and the formation of an SX structure.

##### 3.2.3.2. <001> fcc growth direction relative to the SD (Y–direction) in the XY plane

Varied scan strategies changed the <001> fcc growth direction relative to the SD on the XY plane. The <001> fcc growth direction in the XY plane deviated between 30°–40° relative to the SD (e.g. tracings 1–4 in Figure 6(e)) regardless of the scan strategies. However, several regions in the unidirectionally scanned scan strategy 1 and meander-scanned scan strategy 3 displayed relatively low deviations relative to the SD, such as tracings 5 and 6, as shown in Figure 6(e), respectively. By implementing the meander scanning with an XY–90° scan rotation in scan strategy 4, it was observed that the <001> fcc growth direction in the XY plane was uniformly deviated by 38°–42° relative to the SD (e.g. tracing 4 in Figure 6(e)). This point indicated that the XY–90° scan rotation led to the homogeneous deviation of the <001> fcc growth direction relative to the SD in the XY plane, as will be discussed in the Discussion section.

## 4. Discussion

### 4.1. Epitaxial solidification in the LPBF process

In laser-derived melting processes, e.g. the LPBF process, a high *G/R* ratio is more attributed to the relatively small melt pool than the surrounding powder bed [22]. For melt pools with low depths, such as a flat-top-derived melt pool, the small distance between the melt-pool bottom and melt-pool surface prevents significant undercooling [24]. Additionally, without a high density of solid particles or partially unmelted powders, equiaxed grains do not form from bulk homogeneous nucleation [22]. These conditions favor planar, cellular, or dendritic epitaxial growths, where solidification starts from the melt-pool fusion line and the <001> fcc growth direction is parallel to the direction with the highest thermal gradient [42]. In this study, the texture intensity was>1 MUD, as shown in Figures 4–6. The texture intensity of > 1 MUD indicates the presence of anisotropy [43], which is associated with the epitaxial-growth-structure formation in the LPBF process [44].

To maintain a recurring epitaxial growth between layers, the local thermal-gradient direction of the upper layer is required to align with the <001> fcc directions of the lower layer. If the maximum thermal-gradient direction in the upper layer deviates too far from the <001> fcc direction in the preceding layer, (e.g. induced by different scan strategies layer-by-layer [45]) a new <001> fcc growth occurs in the different thermal-gradient direction [46]. This mismatch between the layers can cause changes in the fcc growth direction and texture, as shown near the boundary between the SX-base and upper parts in the specimens with scan strategy 1 (Figures 4(a) and 5(a)). In the SX-base part in scan strategy 1, the <011>║HD – SD and <001>║BD textures were obtained as shown in Figures 4(a), 5 (a), and 6a.1. It suggested that the <001> fcc growth direction was ║BD in the XZ and YZ planes in the SX-base part. However, the <001> fcc growth direction in the upper part was deviated by 18°–32° relative to the BD (e.g. tracing 2 shown in Figure 5(d)) because a different thermal-gradient direction between the SX-base and upper parts was induced by unidirectional scanning in the upper part.

The maximum misorientation toleration to suppress competitive <001> fcc growth direction between the lower and upper layers has not yet been unraveled [45,47,48]. However, the different thermal-gradient direction caused the growth of new <001> fcc cells in the upper part, e.g. the upper part displayed the <111>║SD and <112>– <111>║HD textures (Figures 4(a) and 5(a), respectively). The changes in the texture did not abruptly occur by equiaxed grain nucleation; however, it gradually transitioned from the epitaxial structure in the layer beneath. This point was discussed by Ishimoto et al. [45]: while epitaxial structure forms from the layer beneath, the <001> fcc direction in the upper layer gradually changes to follow the thermal-gradient direction in the following layers. The preferred gradual transition of the epitaxial growth direction instead of the nucleation of equiaxed grain with different thermal-gradient directions occurs because the energy barrier for epitaxial solidification is smaller than that for equiaxed grain nucleation [49]. The gradual texture change occurred in the transition zone (black arrows in Figures 4(a) and 5(a)) with the difference in the <001> fcc growth direction between the SX-base and upper parts. The higher the difference in the thermal-gradient direction between the upper and lower layers, the shorter the transition region the <001> fcc growth direction changes in [45]. This point highlights that the epitaxial growth was preferred in the LPBF process, although an adjustment in the scan strategy was necessary to control the epitaxial-growth structure and direction.

### 4.2. Mechanism of the SX structure formation by implementing meander scanning and XY–90° scan rotation

The DS process requires a proper grain-selector design for grain selection [18,21]. The absence of the grain selector in this study suggested that the grain selection to obtain an SX structure might be attributed to the scan strategy of the meander scanning with an XY–90° scan rotation.

#### 4.2.1. Meander scanning to promote the <001> fcc growth direction ║BD in the XZ and YZ planes

The <001> fcc growth direction in the XZ plane was closely ║BD in the unidirectionally scanned specimens (tracings 1 and 2 in Figure 4(e)). However, the <001> fcc growth direction on the YZ plane was deviated by 18°–40° relative to the BD (tracings 1 and 2 in Figure 5(e)). This was because of the unidirectional beam movement along the SD axis. Despite the tilting of the growth direction, epitaxial growth occurred to minimize the nucleation energy because the <001> fcc growth direction was aligned layer-by-layer with small misorientations. Changing the scan strategy from unidirectional scanning (scan strategies 1–2) to meander scanning in scan strategy 3 changed the texture and the <001> fcc growth direction relative to the BD on the YZ plane. In scan strategy 3, the <011>║SD and HD textures were observed in the XZ and YZ planes (Figures 4(c) and 5(c), respectively), whereas the <112>– <111>║SD and HD textures formed in scan strategy 2 (Figures 4(b) and 5(b), respectively). This texture change resulted from the decrease in the deviation of the <001> fcc growth direction on the YZ plane from 18°–40° in scan strategies 1–2 (tracings 1 and 2 in Figure 5(e)) to approximately 10°–12° relative to the BD in scan strategy 3 (tracing 4 in Figure 5(e)).

**Figure 7.**
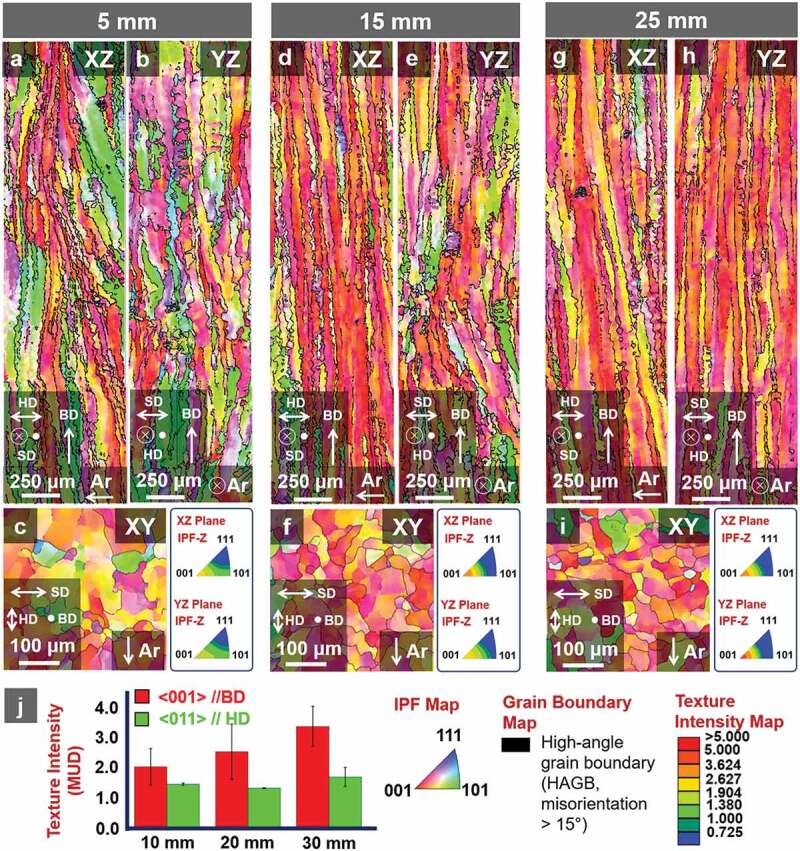
IPF maps in the BD (Z–direction) for the multilayer specimens with scan strategy 3 at different building heights; (a – c) 5 mm, (d – f) 15 mm, and (g – i) 25 mm; (a,d,g) XZ, (b,e,h) YZ, and (c,f,i) XY planes; (j) changes in the <001> and <011> intensities in the BD and HD, respectively; note the re-usage of (i) from Fig. 5c.

The <001> fcc growth direction in the unidirectional scanning was deviated by approximately 30° relative to the BD on the YZ plane, as observed in the solidification structure tilted by approximately 25°–40° relative to the BD in the fusion-track analysis. However, in this study, by implementing the meander scanning, the epitaxial growth on the YZ plane was promoted with the <001> fcc growth closely ║BD, i.e. 10°–12° relative to the BD. This was because the epitaxial growth closely ║BD on the YZ plane was energetically more favorable than causing the nucleation for a new <001> fcc growth direction in the meander scanning. In this case, the <001> fcc growth direction has to be 0° (║BD) or deviated by 45° relative to the BD on the YZ plane [35–37]. The 45° misorientation of the <001> fcc growth direction relative to the BD at each layer creates a perfect 90°- fcc crystal symmetry in the iterant layers in the bidirectional scanning [35]. As for other <001> fcc growth directions on the YZ plane, for example, 30° misorientation relative to the BD causes 120° misorientation of the <001> fcc growth direction between upper and lower layers [35]. At lower building heights, the <001> fcc growth direction on the YZ plane exhibited high misorientation relative to the BD, as indicated by the weak <001>║BD texture (Figure 7(a–c,j)). The epitaxial solidification starts in the first few layers after heterogeneous nucleation at the interface of the molten melt pool and substrate [50]. Therefore, the texture configuration relative to the BD at lower building height is affected by the substrate orientation [51]. A polycrystalline stainless-steel-build substrate was used in this study. As the building height increased to 20 mm (Figure 7(d–f)) and 30 mm (Figure 7(g–i)), the <001>-fcc growth direction on the YZ plane transitioned to closely ║BD as indicated by the stronger <001>║BD texture at high building heights (Figure 7(j)). The texture formation at high building heights is more influenced by the epitaxial growth direction dictated by the temperature distribution in the melt pool [51] than the texture orientation of the stainless-steel-build substrate. In addition, the high building height experienced a low cooling rate from the extensive thermal cycles [52,53]. This caused a higher *G*/*R* ratio and promoted epitaxial growth in the direction of BD. In this study, the epitaxial growth in the direction ║BD was preferred during meander scanning because it was energetically more favorable. Thus, a lower declination of the <001> fcc growth relative to BD on the YZ plane was observed at higher building heights. This study displayed that meander scanning promoted the epitaxial growth of <001> fcc to grow in the direction closely ║BD on the YZ plane.

Thus, implementing meander scanning caused the <001> fcc growth direction in the XZ and YZ planes to be ║BD and promoted the <001>║BD texture formation. Figure 7 displays the IPF maps of the specimen with scan strategy 3 at building heights of 10 (Figure 7(a–c)), 20 (Figures 7(d–f)), and 30 mm (Figures 7(g–i)) on the XZ, YZ, and XY planes. The promotion of the <001> fcc growth direction ║BD was indicated by the grains displaying the <001>║BD texture, particularly in high buildings (Figure 7(g–i)), as well as intensified <001>║BD texture with increasing building heights (Figure 7(j)). However, SX structure formation was yet to be observed by implementing scan strategy 3, suggesting that the XY–90° rotation is requisite to obtain the SX structure.

#### 4.2.2. XY–90° scan rotation to promote the <001> fcc growth direction deviated by 45° relative to the SD in the XY plane

**Figure 8.**
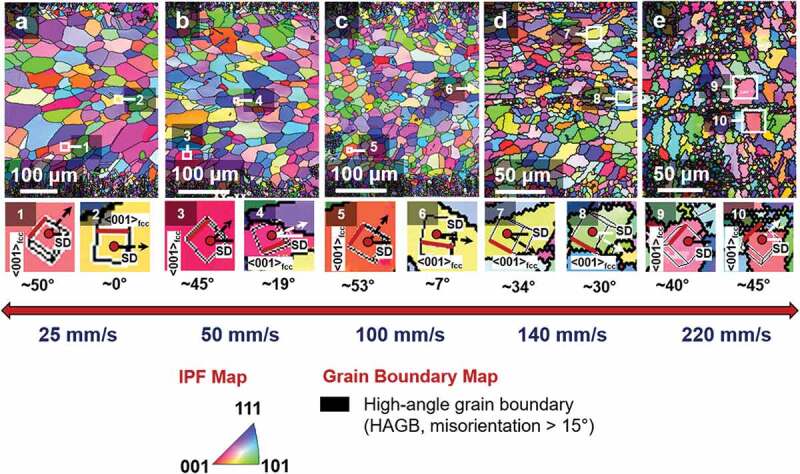
IPF maps in the BD (Z–direction) for the fusion tracks of P 500 W with different scan speeds: (a) 25, (b) 50, (c) 100, (d) 140, and (e) 220 mm/s; note the re-usage of (d) from Fig. 3c. The IPF maps were.

The meander scanning with an XY–90° scan rotation in scan strategy 4 induced a homogeneous <001>║BD texture (Figure 6(d)) and homogeneous <011>║SD and HD textures (Figures 4(d) and 5(d), respectively). Notably, the XY–90° scan rotation in this study differed from the μ-helix 90° scan rotation in the EPBF-derived SX studies [13,15]. The <011>║SD and HD textures indicated that the <001> fcc growth direction in the XY plane was deviated by approximately 45° relative to the SD and HD. The deviation of the <001> fcc growth direction relative to the SD was induced by the beam movement and the circular geometry of the laser beam in the XY plane. Figure 8 shows the IPF maps of the fusion tracks of *P* 500 W with different scan speeds. Under relatively low scan speed (Figure 8(a,b)), the <001> fcc growth in the XY plane exhibited different growth directions relative to the SD between the melt-pool center (e.g. tracings 2 and 4) and edge regions (e.g. tracings 1 and 3). At high speeds (Figure 8(c–e)), the <001> fcc growth direction relative to SD in the XY plane was relatively similar between the melt-pool center and edge. The laser power and scan speed in the track shown in Figure 8(d) are the same as the ones of the multilayer fabrication in this study. The track shown in Figure 8(d) shows that the <001> fcc growth directions in the melt-pool center and edge regions were deviated by approximately 25°–50° relative to the SD in the XY plane. The significantly homogeneous deviation of the <001> fcc growth direction and its deviation close to 45° lead to the fcc cell symmetry on the XY plane. This observation suggested that adjustment in scan speed was necessary to minimize the discrepancies of the <001> fcc growth direction relative to the SD between the melt-pool center and edge in the XY plane.

**Figure 9.**
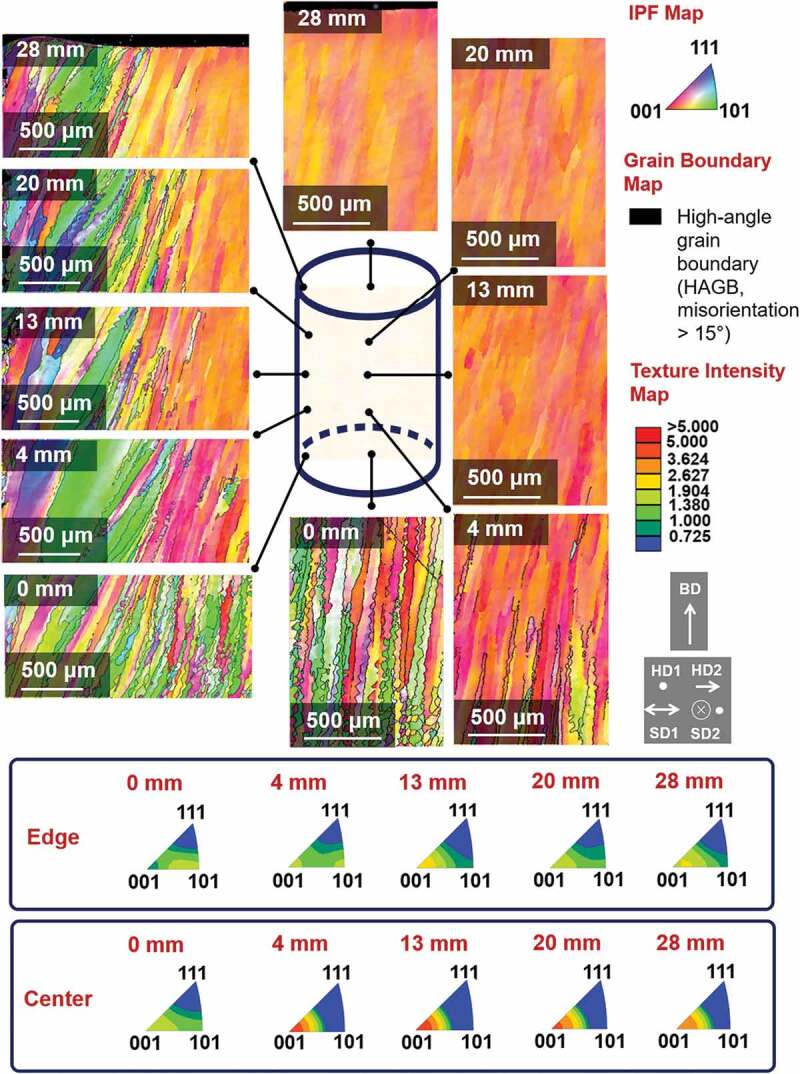
IPF maps in the BD (Z–direction) at the XZ/YZ plane for the specimen with scan strategy 4 at different areas and building heights above the stainless steel build substrate.

**Figure 10.**
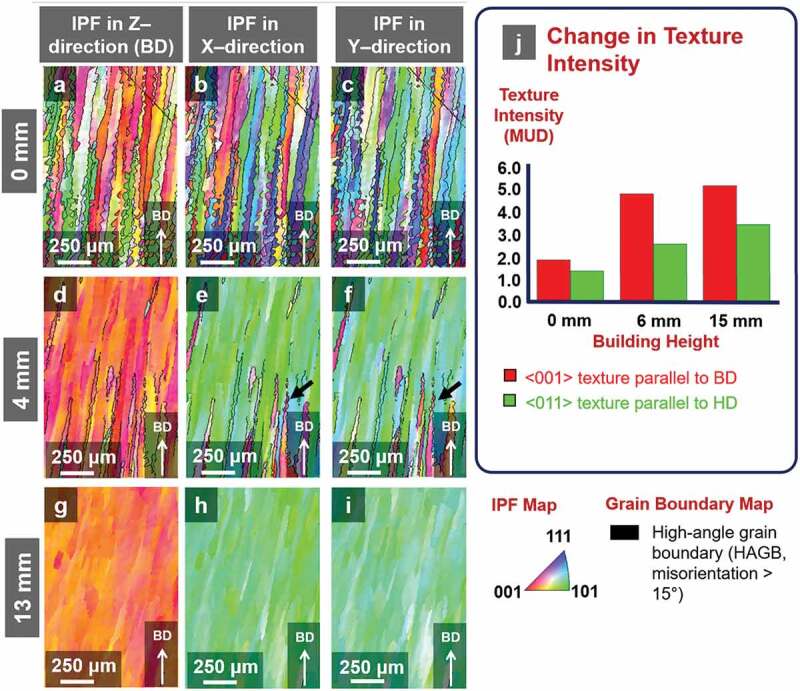
IPF maps at the XZ/YZ plane of the specimen fabricated with scan strategy 4 at different building heights: (a – c) 0 mm, (d – f) 4 mm, and (g – i) 13 mm; the IPF maps orientation were set in the (a,d,g) BD (Z–direction), (b,e,h) HD (X–direction), and (c,f,i) SD (Y–direction); (j) changes in the <001> and <011> intensities at different building heights in the BD and HD, respectively.

Implementing scan strategy 4 caused the formation of the SX structure, as shown in Figure 9. As discussed in section 3.2.3.2, scan strategy 4 caused a more homogeneous <001> fcc growth direction on the XY plane in the deviation range of 38°–42° relative to the SD (tracing 4 in Figure 6(e)). Subsequently, homogeneous <011>║SD and HD textures were observed, particularly at increased building heights (Figure 10(a–i)), which was indicated by the increase in the <011>║HD-texture intensity with increasing building heights, as shown in Figure 10(j).

**Figure 11.**
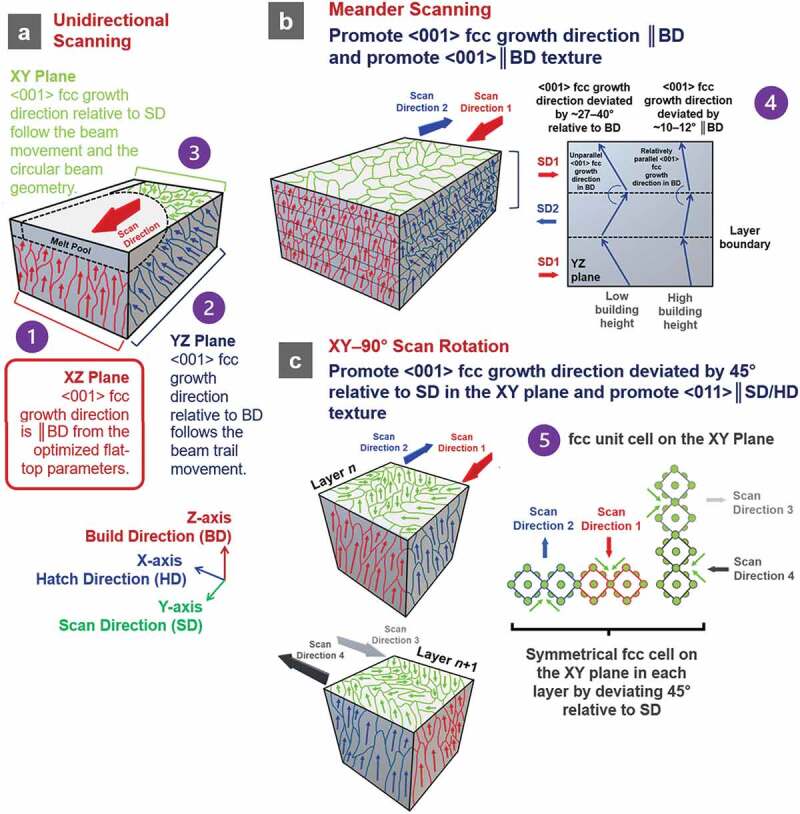
Schematic for the influence of scan strategy on the SX structure formation in this study.

The SX structure formation was dependent on the meander scanning and XY–90° rotation in scan strategy 4, as schematically shown in Figure 11. The flat-top parameter in this study yielded a <001>  fcc growth direction ║BD in the XZ plane (point 1 in Figure 11(a)). With unidirectional scanning, the <001> fcc growth direction in the YZ plane was deviated by 18°–40° relative to the BD (Table 2) attributed to the beam movement in the SD (point 2 in Figure 11(a)). Further, the circular beam geometry caused the deviated <001> fcc growth direction in the XY plane relative to the SD (point 3 in Figure 11(a); Table 2). With meander scanning, two opposite SDs (red and blue arrows in Figure 11(b)) were introduced. Owing to the meander scanning, the epitaxial growth of the <001> fcc growth direction deviated closer to the BD by 10°–12° relative to the BD (point 4 in Figure 11(b)) on the YZ plane. Thus, the meander scanning induced the <001> fcc growth direction to be relatively ║BD in the XZ and YZ planes, leading to <001>║BD texture formation, as shown in Figure 7(j). In scan strategy 4, the meander scanning with an XY–90° scan rotation induced four SDs (red, blue, grey, and black arrows in Figure 11(c)). To promote epitaxial growth in the <001> fcc growth direction along all four SDs on the XY plane, the <001> fcc growth direction in the XY plane must be deviated by 45° relative to the SD to ensure the fcc crystal symmetry in each layer. Point 5 in Figure 11(c) illustrates the symmetrical fcc unit cell on the XY plane with <001> fcc growth direction deviated by 45° relative to the SD. The deviated <001> fcc growth direction caused <011>║SD and HD textures formation, and other textures were eventually overgrown at the increased building height (black arrows in Figure 10(e–f)).

**Table 2.** Change in the <001> fcc growth direction with different scan strategies.

Aspect	Plane	Scan Strategy			
		1	2	3	4
<001> fcc growth direction relative to BD	XZ	4–12°	6–18°	8–11°	10–11°
	YZ	18–32°	27–40°	10–12°	10–11°
<001> fcc growth direction relative to SD	XY	19–40°	25–48°	17–39°	38–42°

**Figure 12.**
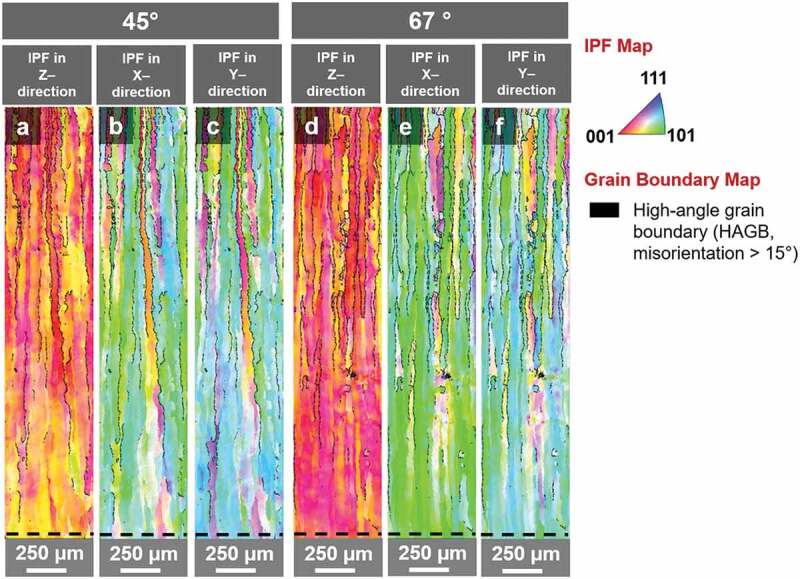
IPF maps of specimens with scan strategies (a – c) 5 and (d – f) 6; the IPF maps orientation were set in the (a,d) BD (Z–direction), (b,e) SD (Y–direction), and (c,f) HD (X–direction); black dashed lines indicate the boundary between the upper and SX-base parts.

For an fcc system, only the 90° scan rotation promoted the homogeneous epitaxial <001> fcc growth on the XY plane as the fcc cells were symmetrical in each layer. The implementation of other scan rotations, e.g. 45° or 67°, failed to form an SX structure, as demonstrated by scan strategies 5 and 6 shown in Figure 12(a–f), respectively. The images in Figure 12(a,d) show the IPF maps parallel to the BD (Z – direction), whereas Figure 12(b,c,e,f) display the IPF maps parallel to the X- and Y-axes. The <001>║BD textures shown in Figure 12(a,d) indicate the <001> fcc growth direction was almost ║BD. However, these <001> fcc growth directions ║BD were induced by the meander scanning in scan strategies 5 and 6. In contrast, the inhomogeneous texture formations shown in Figure 12(b,c,e,f) suggested that the <001> fcc growth direction relative to the SD on the XY plane was not uniform. Thus, in addition to optimizing the laser power and scan speed, meander scanning and XY–90° scan rotation were necessary to obtain the <001> fcc growth direction ║BD in the XZ and YZ planes, as well as the <001> fcc growth direction deviated by approximately 45° relative to the SD in the XY plane, to achieve a flat-top-LPBF-derived SX structure.

## 5. Conclusion

The influence of the scan strategy on pure Ni SX structures in the flat-top-LPBF was examined. In this study, implementing a meander scanning with an XY–90° scan rotation was necessary to obtain the flat-top-derived SX structure. The meander scanning promoted epitaxial <001> fcc growth in the direction ║BD, causing the formation of the <001>║BD texture. Meanwhile, the XY–90° scan rotation caused epitaxial <001> fcc growth in the direction deviated by approximately 45° in the XY plane. The deviation of 45° relative to the SD in the XY plane, which caused the formation of the <011>║SD and HD textures, was attributed to the laser beam movement with the circular beam geometry on the XY plane. Thus, this study highlighted the significance of meander scanning with an XY–90° scan rotation to obtain a flat-top-LPBF-derived SX structure.

## Data Availability

The data in this study cannot be shared as part of other ongoing studies.
